# Immunomodulatory Activity of *Lactococcus lactis* GCWB1176 in Cyclophosphamide-Induced Immunosuppression Model

**DOI:** 10.3390/microorganisms8081175

**Published:** 2020-08-02

**Authors:** Sun Woo Jin, Gi Ho Lee, Min Jung Jang, Gyeong Eun Hong, Jae Young Kim, Gi Deok Park, Hui Jin, Hyun Su Kim, Jae Ho Choi, Chul Yung Choi, Su Gwon Lee, Hye Gwang Jeong, Yong Pil Hwang

**Affiliations:** 1 College of Pharmacy, Chungnam National University, Daejeon 34134, Korea; mpassword@cnu.ac.kr (S.W.J.); ghk1900@cnu.ac.kr (G.H.L.); 2 Department of Research, GREEN CROSS Wellbeing Co., Ltd., Seongnam 13595, Korea; mjjang@gccorp.com (M.J.J.); hge0121@gccorp.com (G.E.H.); jaeyoung4740@gccorp.com (J.Y.K.); pkd6300a@gccorp.com (G.D.P.); iuhnij@gccorp.com (H.J.); hs_kim4@gccorp.com (H.S.K.); 3 Subtropical/Tropical Organism Gene Bank, Jeju National University, Jeju 63243, Korea; chlkoala@naver.com; 4 Jeonnam Bioindustry Foundation, Jeonnam Institute of Natural Resources Research, Jeollanamdo 59338, Korea; blockstar@hanmai.net; 5 College of Pharmacy, Gachon University, Incheon 21936, Korea; clud159@naver.com; 6 Department of Pharmaceutical Engineering, International University of Korea, Jinju 52833, Korea; 7 Fisheries Promotion Division, Mokpo City, Jeollanamdo 58613, Korea

**Keywords:** *Lactococcus lactis* subsp. *lactis* GCWB1176, immunostimulatory activity, cyclophosphamide, NK cell activity, macrophage phagocytosis

## Abstract

Recently, *Lactococcus lactis* subsp. *lactis* has been reported to have immunostimulating properties in an immunosuppressed-animal model. However, the immunological activities of *Lactococcus lactis* and the molecular mechanisms remain unclear. In this report, we evaluated the immunostimulating activity and associated mechanisms of *Lactococcus lactis* subsp. *lactis* GCWB1176 (GCWB1176) in macrophages and cyclophosphamide (CTX)-induced immunosuppressed mice. In a series of safety tests, GCWB1176 was found to have a negative response to hemolysis, as well as susceptibility to antibiotics. Administration of GCWB1176 elevated natural killer (NK) cell activities; concanavalin A-induced T cell proliferation; and serum levels of tumor necrosis factor (TNF)-α, interferon (IFN)-γ, interleukin (IL)-2, IL-4, IL-10 and IL-12 in CTX-induced immunosuppressed mice. In RAW264.7 macrophages, treatment with GCWB1176 induced phagocytic activity and increased the production of nitric oxide (NO) and expression of inducible NO synthase. Simultaneously, GCWB1176 increased the production of TNF-α, IFN-γ, IL-1β, IL-10 and IL-12 from mouse splenocytes and RAW264.7 cells. In addition, GCWB1176 significantly increased the transcriptional activities of NF-κB and iNOS. Taken together, GCWB1176 improved immune function through the activation of macrophages and NK cells. These findings suggest that dietary supplementation of GCWB1176 may be used to enhance immunity.

## 1. Introduction

The mammalian immune system consists of innate and adaptive immunity [1]. Among them, innate immunity plays a role in recognizing a deviation from homeostasis due to infectious or non-infectious assaults; adaptive immunity, including humoral immunity and cell-mediated immunity; killing infected cells; and neutralizing antigens [1,2]. Innate immunity is mediated by phagocytes including macrophages and neutrophils and is an important component of host defense against invading microbial pathogens [3,4].

Lymphocytes and macrophages play an important role in innate and adaptive immunity [5]. The activation of lymphocytes and macrophages causes the production of inflammatory cytokines and mediators, such as reactive oxygen species (ROS) and nitric oxide (NO), and the secretion of proinflammatory cytokines, such as tumor necrosis factor-α (TNF-α), interferon-(IFN)-γ, interleukin (IL)-6 and IL-12 [6,7,8,9]. These molecules are involved in the mitogen-activated protein kinase (MAPK) and the nuclear factor-κB (NF-κB) signaling pathways. NO is an important intracellular and intercellular regulatory molecule with many physiological functions. It is synthesized by inductive nitric oxide synthase (iNOS) and has many physiological roles, including immune response, vascular dilatation, neurotransmission and platelet aggregation inhibition [7].

Loss of homeostasis in the immune system affects the immune response and leads to a variety of diseases [10]. Cyclophosphamide (CTX) is a well-known alkylating cytotoxic drug that is effective in organ transplantation, the treatment of cancer and autoimmune diseases in part because of its ability to modulate immune responses [11,12]. CTX is one of the major immunomodulatory drugs used in clinical trials, which damages the DNA of normal cells and has immunosuppressive effects [13]. However, immunomodulators, especially drugs containing single ingredients, can cause side effects such as fever, headache, hypertension and neurotoxicity. Immunosuppression can be fatal and is damaging to the immune system of the organism. Therefore, safe immunoregulatory agents that can prevent immunodeficiency in cancer patients receiving chemotherapy have been investigated, including dietary supplements and functional foods that promote health [14].

The use of probiotics for improving immune health status in immunodeficient patients has gained a special interest in recent years [15]. Probiotics are commonly used in various products because of the medically confirmed functions such as beneficial bacterial proliferation, harmful bacteria suppression, improvement of intestinal health, blood cholesterol reduction, immunity enhancement, endogenous infection suppression and anti-cancer effects [16,17]. Probiotics are used for the treatment and prevention of various diseases, but they have not been widely used due to a lack of understanding of the mechanism of action. In addition, although the immuno-promoting efficacy of lactic acid bacteria (LAB) has been actively studied, the efficacy of some specific strains have not been characterized; thus, further studies on various strains and the mechanisms of action are required [18]. Among the 13 genera of LAB, *Lactococcus*, *Lactobacillus*, *Leuconostoc* and *Enterococcus* are known to play key roles in improving immunity, maintaining intestinal microbial balance and preventing gastrointestinal infections [19,20,21,22,23]. Recent studies have shown that selenium exopolysaccharide produced by *Lactococcus lactis* subsp. *lactis* has antioxidant and immunomodulatory functions in immunosuppressive animal models [24]. However, no studies have been reported on the mechanism of action related to the immunostimulating efficacy of *Lactococcus lactis* in CTX-induced immunosuppressed mice.

In this study, we investigated the immunostimulating effects and associated mechanisms of *Lactococcus lactis* subsp. *lactis* GCWB1176 isolated from mozzarella cheese in mouse macrophages and CTX-induced immunosuppressed mice.

## 2. Materials and Methods

### 2.1. Materials

Roswell Park Memorial Institute (RPMI) 1640 medium, Dulbecco’s Modified Eagle’s Medium (DMEM), fetal bovine serum (FBS), streptomycin and penicillin were purchased from Life Technologies (Carlsbad, CA, USA). Enzyme-Linked immunosorbent assay (ELISA) kits for IL-1β, IL-2, IL-4, IL-10, IL-12p70, TNF-α and IFN-γ were from R&D Systems (Minneapolis, MN, USA). 3-(4,5-Dimethylthiazol-2-yl)-2,5-diphenyltetrazolium bromide (MTT) was from USB Corp. (Cleveland, OH, USA) and the lactate dehydrogenase (LDH) release detection kit was from Roche Applied Science (Indianapolis, IN, USA). All kits were used according to the manufacturer’s protocols. CTX monohydrate, lipopolysaccharide (LPS), concanavalin (Con)A and dimethyl sulfoxide (DMSO) were from Sigma-Aldrich (St. Louis, MO, USA). YAC-1 cells, a mouse lymphoma cell line, and RAW264.7 cells, a mouse macrophage cell line, were obtained from ATCC (Manassas, VA, USA; no. ATCC^®^TIB-160^TM^ and ATCC^®^TIB-71^TM^). Antibodies for β-actin, iNOS, NF-κB-p65 and secondary antibody (HRP-linked anti-rabbit IgG) were obtained from Santa Cruz Biotechnology (Santa Cruz, CA, USA). piNOS-Luc and pNF-κB-Luc were purchased from Addgene (Watertown, MA, USA).

### 2.2. Microbial Strain and Culture Conditions

*Lactococcus lactis* subsp. *lactis* GCWB1176 was isolated from mozzarella cheese and filed by GREEN CROSS Wellbeing (GCWB), Seongnam, Korea. This strain is deposited in the Korea Culture Center of Microorganisms under registration number KCCM12687P. *Lactococcus lactis* subsp. *lactis* GCWB1176 was cultured in optimized medium under anaerobic conditions. The strain was cultured in De Man, Rogosa and Sharpe (MRS) broth (Sigma-Aldrich, St. Louis, MO, USA) under anaerobic conditions at 37 °C for 24 h. The bacterial cells of stationary phase were centrifuged at 8000× *g* for 20 min at 4 °C. The cell pellet was washed twice with sterile phosphate-buffered saline (PBS) buffer (Life Technologies, Carlsbad, CA, USA) and adjusted to the appropriate density in DMEM or RPMI1640 medium containing 10% FBS 20 mM 4-(2-hydroxyethyl)-1-piperazineethanesulfonic acid (HEPES) (Life Technologies, Carlsbad, CA, USA) for the in vitro experiments or in sterile PBS for the in vivo experiments.

### 2.3. Measurement of Live Cells

The numbers of live GCWB1176 (1 × 10^11^ CFU/g freeze-dried lactobacilli) were quantified by a 10-fold serial dilution and the plate-count method of MFDS (Ministry of Food and Drug Safety). Cell density during culturing in either the batch culture or MBR was analyzed by real-time measurement of optical density at 660 nm (G10S UV-Vis; Scinco Co., Gangnam, Korea).

### 2.4. Safety and Antimicrobial Susceptibility Testing (E-Test)

For the hemolysis test, *Lactococcus lactis* subsp. *lactis* GCWB1176 was plated on blood agar plates, containing 5% (*w*/*v*) sheep blood (Synergy Innovation, Seoul, Korea), and incubated for 18 h at 37 °C. Blood agar plates were examined for signs of β-hemolysis (clear zones around colonies), α-hemolysis (green-hued zones around colonies), or γ-hemolysis (no zones around colonies). For Lactococcus strains, the minimum inhibitory concentrations (MICs) (μg/mL) of nine antibiotics were determined using commercial E-test (Liofilchem MIC test Strip, Roseto degli Abruzzi, Italy): ampicillin, vancomycin, gentamicin, kanamycin, streptomycin, erythromycin, clindamycin, tetracycline and chloramphenicol. Bacterial suspensions with a turbidity equivalent to McFarland standard 1 were swabbed evenly onto MRS agar (BD DIFCO, 288210) plates with a sterile cotton swab. After drying the surfaces of the plates, the E-test strips (AB Biodisk, Solna, Sweden) of all antimicrobial agents tested (0.016–256 μg/mL) were applied. E-test strips were placed on the surface of the inoculated agar and incubated at 37 °C for 24 h. MICs were read directly from the test strip according to the instructions of the manufacturer. The concentration-dependent susceptibility was determined according to the European Food Safety Authority (EFSA) [25].

### 2.5. Cell Culture and Sample Treatment

Mouse splenocytes, YAC-1 cells and RAW264.7 macrophages were cultured in RPMI 1640 and DMEM medium containing 10% FBS, streptomycin sulfate and penicillin at 37 °C and 5% CO_2_. Cells were treated with various concentrations (1.65 × 10^4^–1.65 × 10^6^ cfu/mL) of GCWB1176, LPS (10 ng/mL) and ConA (1 μg/mL) for various times.

### 2.6. Isolation of Splenocytes

The spleen tissues were aseptically extracted from the ICR mouse (six-week-old male, 20 ± 2 g). The tissues were disaggregated via passage through a 70 μm nylon mesh (Becton-Dickinson, Bedford, MA, USA) in RPMI-1640 medium (Life Technologies, Carlsbad, CA, USA) and the cells were purified via centrifugation at 450× *g* for 5 min and red blood cells (RBC) were removed with ACK lysis buffer (Sigma-Aldrich, St. Louis, MO, USA). Splenocytes were then washed with PBS, centrifuged at 1000× *g* for 5 min and suspended in RPMI containing 10% FBS, penicillin and streptomycin sulfate.

### 2.7. Measurement of Cell Cytotoxicity and Proliferation

Cell viability was assessed with the MTT assay or the LDH cytotoxicity assay. RAW264.7 mouse macrophage cells (1 × 10^6^ cells/well) were seeded in 96-well plates containing 100 µL RPMI 1640 medium and 10% FBS and then incubated for 24 h. GCWB1176 (1.65 × 10^4^–1.65 × 10^6^ cfu/mL) was added, followed by incubation for 24 h. MTT solution (5 mg/mL in PBS) was added for 30 min at 37 °C and 5% CO_2_; the medium was then discarded and formazan crystals were dissolved with 200 µL DMSO, and the absorbance of each well at 550 nm was measured with a microplate reader (Varioskan; Thermo Fisher Scientific, Waltham, MA, USA). Cell proliferation was examined with a water-soluble tetrazolium (WST)-1 assay kit according to the manufacturer’s instructions. Briefly, mouse spleen cells (1 × 10^6^ cells/well) were seeded in 96-well plates in DMEM with 10% FBS. After mixing with 10 µL RPMI 1640 medium (negative control) or 10 µL of 200 μg/mL ConA (positive control), the cells were incubated at 37 °C and 5% CO_2_ for 48 h. The supernatant was removed, and the cells were used in the WST-1 assay. Relative cytotoxicity was quantified by measuring the absorption at 550 nm with a microplate reader.

### 2.8. Preparation of the Cyclophosphamide-Induced Immunosuppression Model in Mice

**Figure 1:**
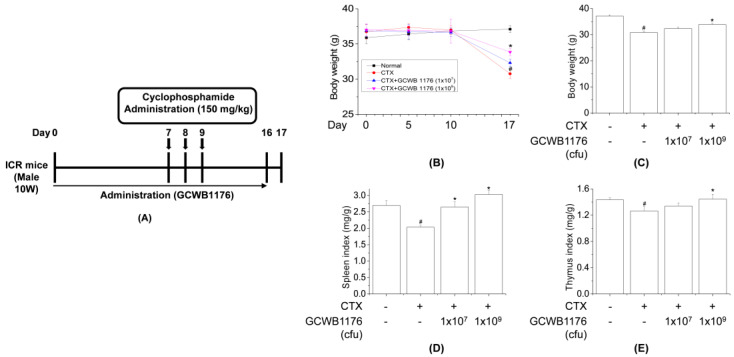
Schematic diagram of the experimental protocol in mice (**A**). Effect of orally administered GCWB1176 on body weight changes (**B**,**C**), spleen index (**D**), and thymus index (**E**) in cyclophosphamide (CTX)-immunosuppressed mice. GCWB1176 (1 × 10^7^ or 1 × 10^9^ cfu/day) suspended in saline was administered orally once daily for 16 days. Mice were treated on Days 7–9 by intraperitoneal injection of cyclophosphamide (CTX, 150 mg/kg/day) in a total volume of 100 µL of saline. Normal control group was treated with vehicle alone. The immune organ index (%) was calculated according to the formula: Index = organ weight (mg)/body weight (g). Thymuses and spleens were obtained on Day 17 and weighed. Data are expressed as mean ± SD (*n* = 8). # *p* < 0.001 vs. normal group. * *p* < 0.001 vs. CTX treated group.

Ten-week-old male ICR mice (30 ± 2 g) were obtained from Samtako (Osan, Korea) and housed in a room with controlled temperature (22 °C ± 2 °C) and humidity (50% ± 5%) on a 12:12-h light/dark cycle with free access to food and water. The mice used in this study were handled in accordance with the Guidelines for the Care and Use of Laboratory Animals published by the U.S. National Institutes of Health (NIH Publication NO. 85-23, 1996), and all experimental procedures were approved by the Committee on Ethics of Animal Experiments of International University of Korea (Ethics NO, IUK-2019-10-1). ICR mice were divided into four groups (*n* = 8), each consisting of normal, cyclophosphamide (CTX), CTX+GCWB low (1 × 10^7^ cfu) and CTX+GCWB high (1 × 10^9^ cfu). In total, 24 mice were injected intraperitoneally (i.p.) with CTX in sterile saline (150 mg/kg) for 3 consecutive days to establish immunosuppressed models (Figure 1). GCWB1176 in saline was administered intragastrically at 1 × 10^7^ cfu and 1 × 10^9^ cfu once daily for 16 days. Mice were treated on Days 7–9 by intraperitoneal (i.p.) injection of cyclophosphamide (CTX, 150 mg/kg/day) in a total volume of 100 µL of saline. For the measurement of body and spleen weights, mice were weighed on Days 0 and 17. At the end of the experiment, the mice were sacrificed by injection of 200 mg/kg pentobarbital, and organs including the spleen were immediately removed and weighed. The immune organ index (%) was calculated according to the formula: Index = organ weight (mg)/body weight (g).

### 2.9. Assay for Macrophage Phagocytosis

The phagocytosis assay was performed as previously described [26]. Briefly, 100 µL fluorescein-5-isothiocyanate (FITC)-labeled *Escherichia coli* (Molecular Probes, Eugene, OR, USA) were added to the wells of a 96-well plate containing RAW264.7 macrophages. The plate was incubated for various times at 37 °C in a humidified atmosphere of 5% CO_2_. Extracellular fluorescence was quenched by adding 100 mL Trypan blue. After 1 min, FITC-labeled bacteria that had not been phagocytosed by macrophages were washed away, and the macrophages were rinsed twice with PBS and then lysed with lysis buffer (10 mM Tris–HCl [pH 7.5], 130 mM NaCl, 1% Triton X-100, 10 mM Na_2_HPO_4_ and 10 mM Na_4_P_2_O_7_). The relative fluorescence intensity of bacteria inside the macrophages was determined at excitation and emission wavelengths of 480 and 520 nm, respectively, using a microplate reader. The relative phagocytic activity was calculated as the percent fluorescence intensity of sample-supplemented vs. unsupplemented (control) FITC-labeled bacteria.

### 2.10. Assay of NK Cell Activity

The spleen tissues were aseptically extracted from each mouse and ground into a single-cell suspension using sterile gauze and washed three times with RPMI 1640 medium. Cells were centrifuged at 1000× *g* for 5 min at room temperature and 100 µL of spleen cell suspension (1 × 10^6^ cells/well) were seeded in a 96-well cell culture plate with 1 × 10^5^ NK-sensitive YAC-1 cells at an effector cell:target cell ratio of 10:1, while 100 µL RPMI 1640 medium were used as a control. After 4-h incubation at 37 °C (5% CO_2_), the plate was centrifuged at 800× *g* for 5 min and the culture supernatant (100 µL/well) was mixed with LDH solution (Promega, Madison, WI, USA); the absorbance of each well was measured at 490 nm. NK cell cytotoxicity was calculated using the following formula: Cytotoxicity (%) = [(experimental release−spontaneous release)/(maximum release − spontaneous release)] × 100.

### 2.11. Measurement of Cytokine Levels

The cytokine levels in blood samples or cell culture media were quantified using ELISA kits, according to the manufacturer’s instructions. Briefly, RAW264.7 cells and splenocytes were cultured for 3 or 30 h at a density of 5 × 10^5^ cells/well in 96-well plates. Supernatants were removed at the indicated times and cytokine (IL-1β, IL-2, IL-4, IL-10, IL-12p70, TNF-α and IFN-γ) production was analyzed based on sandwich immunoassays, as per the manufacturer’s protocol (R&D Systems).

### 2.12. Nitrite Assay

RAW264.7 cells (5 × 10^5^ cells/well) were seeded in 96-well plates containing 200 µL RPMI1640 medium and 10% FBS and then incubated overnight. Cells were treated with various concentrations of GCWB1002 (1.65 × 10^4^ and 1.65 × 10^5^ cfu/mL) for 48 h. The cell culture media were collected, and nitrite was measured using Griess reagent. Equal volumes of Griess reagent (1:1 of 1% sulfanilamide in 5% phosphoric acid and 0.1% N-1 naphthylethylenediamine in 5% phosphoric acid) and sample were incubated together at room temperature for 5 min. Absorbance at 550 nm was measured using a microplate reader (Varioskan; Thermo Fisher Scientific, Waltham, MA, USA).

### 2.13. Western Blotting

RAW264.7 macrophages were treated with LPS (10 ng/mL) or GCWB1176 for 24 h or 1 h, and the protein levels of iNOS and NF-κB-p65 were determined immunochemically using specific antibody. The total protein or nuclear protein concentration of supernatants was estimated using the Bradford method and 50 μg protein was separated with 10% SDS-PAGE and then transferred onto polyvinylidene difluoride membranes. RAW264.7 macrophages were cultured with GCWB1176. Total cellular protein (50 μg) was then resolved by 10% SDS–PAGE and transferred onto polyvinylidene difluoride membranes. After blocking, the membranes were incubated with target antibody. Horseradish peroxidase-conjugated secondary antibody to IgG was used. The blots were probed using the ECL Western blot detection system, as instructed by the manufacturer.

### 2.14. Statistical Analysis

Results are expressed as mean values ± standard deviation (SD) of triplicate experiments. Data from the animal study are expressed as mean ± SD (*n* = 8). Mean differences were evaluated by analysis of variance followed by Dunnett’s post hoc test, and *p* values < 0.05 were considered statistically significant.

## 3. Results

### 3.1. Lactococcus lactis GCWB1176 Is a Safe Strain of Probiotics

To evaluate whether GCWB1176 is safe for probiotic use, we performed a hemolysis test using *E. coli* ATCC 25922, *Staphylococcus aureus* ATCC12600 and *Enterococcus faecalis* ATCC19433 as a positive control. GCWB1176 or *Enterococcus faecalis* ATCC19433 were found to have a negative response gamma hemolysis activity (Table 1), while *E. coli* ATCC 25922 and *Staphylococcus aureus* ATCC12600 showed alpha and beta hemolysis activity. To evaluate the susceptibility of GCWB1176 to the selected antibiotics, we performed an assay for determining the MICs. As shown in Table 2, GCWB1176 was sensitive to the selected antibiotics compared to breakpoints of European Food Safety Authority (EFSA). Thus, our results demonstrate that GCWB1176 is safe for use as a probiotic in industrial applications.

### 3.2. The Effect of GCWB1176 on Body Weight and Organ Indices

To investigate the immune-enhancing effects of GCWB1176 under physiological conditions, we performed an in vivo study (Figure 1A). The spleen and thymus are important organs involved in specific and nonspecific immunity and the indices of spleen and thymus reflects immune function and prognosis. Generally, the enhancement of immune stimulation induces an increase in spleen tissue [13]. GCWB1176 in saline was administered intragastrically (i.g.) at 1 × 10^7^ and 1 × 10^9^ cfu once daily for 16 days. Mice were treated on Days 7–9 by intraperitoneal injection of cyclophosphamide (CTX, 150 mg/kg/day) in a total volume of 100 µL of saline. Body weight, spleen and thymus indices of CTX-treated mice were significantly reduced compared with normal groups (vehicle-treated controls), indicating proper functioning of the immunosuppressed model. Furthermore, the body weight, spleen and thymus indices of the GCWB1176-treated group (1 × 10^7^ and 1 × 10^9^ cfu, i.g.) were dose-dependently increased compared with the CTX-treated group (Figure 1B–E). These results indicate that GCWB1176 enhance immunity in immunosuppressed model mice.

### 3.3. Effects of GCWB1176 on Cytokine Levels, NK Cell Activity, and Lymphocyte Proliferation in a Mouse Model of CTX-Induced Immunosuppression

**Figure 2:**
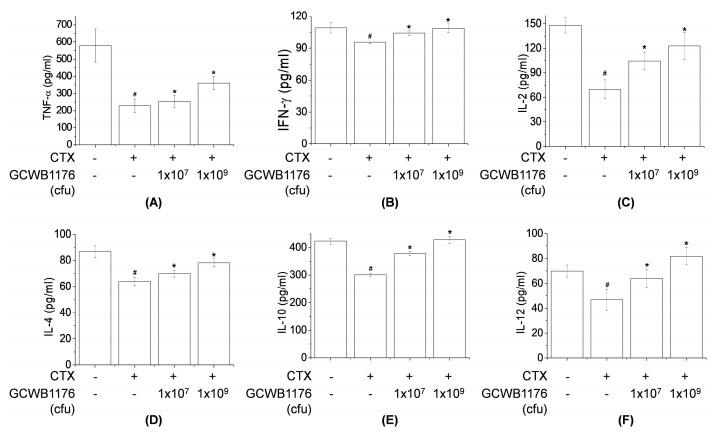
Effect of orally administered GCWB1176 on levels of blood cytokines in CTX-immunosuppressed mice: TNF-α (**A**); IFN-γ (**B**); IL-2 (**C**); IL-4 (**D**); IL-10 (**E**); and IL-2 (**F**). GCWB1176 (1 × 10^7^ or 1 × 10^9^ cfu/day) suspended in saline was administered orally once daily for 16 days. Mice were treated on Days 7–9 by intraperitoneal injection of cyclophosphamide (CTX, 150 mg/kg/day) in a total volume of 100 µL of saline. Normal control group was treated with vehicle alone. Cytokines were assayed using commercial ELISA kits. Data are expressed as mean ± SD (*n* = 8). # *p* < 0.001 vs. normal group. * *p* < 0.001 vs. CTX treated group.

**Figure 3:**
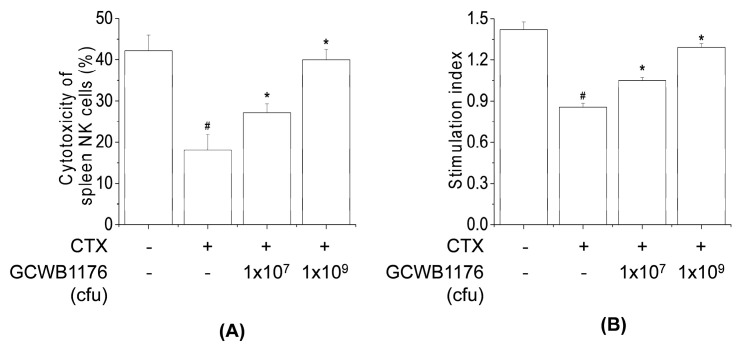
Effect of orally administered GCWB1176 on NK cell activity and ConA-induced T cell proliferation in CTX-immunosuppressed mice. Cyclophosphamide-induced immunosuppressive ICR mice were treated with GCWB1176 (1 × 10^7^ or 1 × 10^9^ cfu/day) for 16 days, and the effects of GCWB1176 on NK cell activity (**A**) and ConA-induced T cell proliferation (**B**) were detected. Data are expressed as mean ± SD (*n* = 8). # *p* < 0.001 vs. normal group. * *p* < 0.001 vs. CTX treated group.

Activated macrophages release various immunomodulatory cytokines such as TNF-α, IFN-γ, and interleukins, which are potent immune modulators [27]. To investigate the effects of GCWB1176 on CY-induced immunosuppression, cytokines were quantified in serum. As shown in Figure 2, serum levels of TNF-α, IFN-γ, IL-2, IL-4, IL-10 and IL-12 were significantly lower in the CTX-treated group than in normal groups. Furthermore, cytokine levels (TNF-α, IFN-γ, IL-2, IL-4, IL-10 and IL-12) in serum of CTX plus GCWB1176-treated group (1 × 10^7^ and 1 × 10^9^ cfu, i.g.) were dose-dependently increased compared to the CTX-treated group (Figure 2). NK cells are the first line of defense against tumor cells and virally infected cells [28]. As shown in Figure 3, CTX significantly inhibited NK cell activity and T lymphocyte proliferation (Figure 3). Compared with the CTX-treated group, all doses of GCWB1176 enhanced NK cell activity and lymphocyte proliferation (Figure 3). These results indicate that GCWB1176 can alleviate CTX-induced immunosuppression.

### 3.4. Effects of GCWB1176 on Viability and Macrophage Phagocytosis

**Figure 4:**
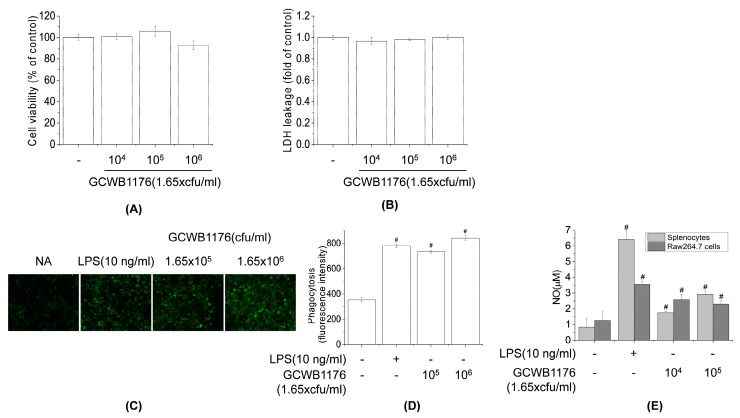
Effects of GCWB1176 on phagocytic activity, NO production in macrophages. Cells were treated with various concentrations of GCWB1176 (1.65 × 10^4^–1.65 × 10^6^ cfu/mL) for 24 h. (**A**) The cell viability was determined by MTT assays. (**B**) Cell cytotoxicity was determined based on LDH release assays. Cells were treated with various concentrations of GCWB1176 (1.65 × 10^5^–1.65 × 10^6^ cfu/mL) for 24 h. After FITC-labeled *E. coli* were added, the cells were incubated for 2 h. The cell culture media containing unphagocytosed FITC-labeled *E. coli* were removed, and fluorescence was measured with a fluorescence microplate reader (**C**). A fluorescence microscope image of RAW264.7 cells was stained with FITC-labeled E. coli for 2 h (**D**). Mouse splenocytes and RAW264.7 cells were treated with various concentrations of GCWB1176 (1.65 × 10^4^–1.65 × 10^5^ cfu/mL) for 24 h. Supernatants were harvested 48 h later and assayed for NO (**E**). All data are expressed as the mean ± SD of three independent experiments. # *p* < 0.001 vs. control group.

To determine the immune-enhancing effect effects of GCWB1176, we first determined the cytotoxic effects in *RAW264.7* cells following treatment with various GCWB1176 (1.65 × 10^4^–1.65 × 10^6^ cfu/mL) concentrations. The cytotoxicity and cell viability of GCWB1176 to RAW264.7 cells were evaluated using MTT (Figure 4A) and LDH (Figure 4B) assays at 24 h. GCWB1176 did not affect the viability of RAW264.7 cells up to concentrations of 1.65 × 10^6^ cfu/mL. These results show that GCWB1176 has no cytotoxicity towards RAW264.7 cells. Concentrations of less than 1.65 × 10^6^ cfu/mL of GCWB1176 were used for subsequent experiments to evaluate their immunostimulatory activities.

Phagocytosis is very important for innate and acquired immune systems since it plays a role in defense against pathogens, chronic inflammation and promotion of tissue repair [29]. Activation of phagocytosis is caused by stimulation of Fc receptors on the surfaces of monocytes, macrophages and neutrophils. These immune cell activations induce a wide range of immune responses such as cytokine synthesis, Ab-dependent cellular cytotoxicity, antibody-dependent cytotoxicity and the production of ROS [30]. The immune-enhancing effect of GCWB1176 on macrophage phagocytosis was measured by internalization of FITC-labeled *E. coli* particles using a fluorescence spectrometer and fluorescence microscopy. As shown in Figure 4C, the fluorescence intensity after taking up FITC-labeled *E. coli* was significantly enhanced in cells after treatment with GCWB1176 or LPS for 24 h, compared with untreated RAW264.7 macrophages (Figure 4C). In addition, green fluorescence was observed in RAW264.7 macrophages using fluorescence microscopy, which can provide visual evidence of phagocytic uptake. The intensity of green fluorescence in GCWB1176 or LPS-treated cells was higher than in untreated RAW264.7 macrophages after taking up FITC-labeled *E. coli* (Figure 4D). These results suggest that GCWB1176 can effectively promote the phagocytic capabilities of macrophages.

To further investigate whether GCWB1176 activates macrophages, we measured NO production in mouse splenocytes and RAW264.7 macrophages incubated with GCWB1176 for 48 h by measuring NO concentrations in the culture supernatant by the Griess reaction. GCWB1176 (1.65 × 10^4^ and 1.65 × 10^5^ cfu/mL) increased NO levels in a concentration-dependent manner (Figure 4E).

### 3.5. Effects of GCWB1176 on Cytokine Productions in Mouse Splenocytes and RAW264.7 Macrophages

**Figure 5:**
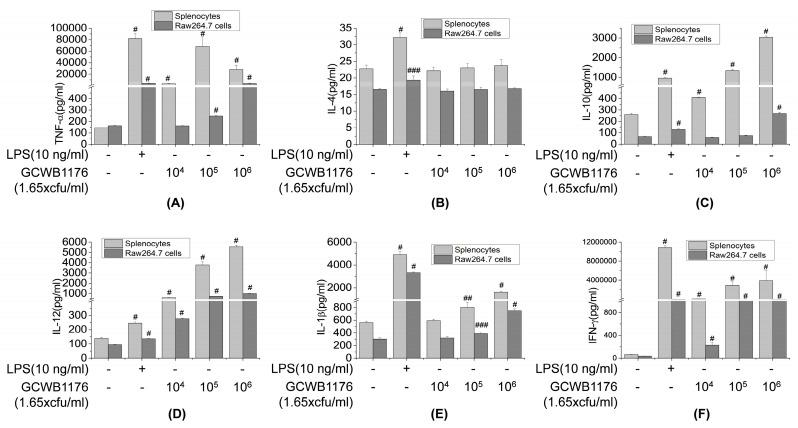
Effects of GCWB1176 on macrophage-related cytokine production in Mouse splenocytes and RAW264.7 macrophages. Cells were treated with various concentrations of GCWB1176 (1.65 × 10^4^–1.65 × 10^6^ cfu/mL) for 3 or 24 h. The amounts of TNF-α (**A**), IL-4 (**B**), IL-10 (**C**), IL-12 (**D**), IL-1β (**E**), and IFN-γ (**F**) released into the culture medium were measured by ELISA kit. # *p* < 0.001 vs. control group. ## *p* < 0.01 vs. control group. ### *p* < 0.05 vs. control group.

To evaluate the role of the GCWB1176 in macrophage functions, GCWB1176 were tested for TNF-α, IFN-γ, IL-1β, IL-4, IL-10 and IL-12 production in RAW264.7 cells and splenocytes. The levels of these cytokines secreted into the culture supernatants were quantified by ELISA. As shown in Figure 5, GCWB1176 increased TNF-α, IFN-γ, IL-1β, IL-4, IL-10 and IL-12 in a concentration-dependent manner (Figure 5). These data demonstrate that GCWB1176 is a potent inducer of cytokine secretion.

### 3.6. Effects of GCWB1176 on NF-κB and iNOS Activation in RAW264.7 Macrophages

Because NO is synthesized by iNOS, we explored the effects of GCWB1176 on iNOS expression in RAW264.7 cells using Western blotting and luciferase reporter gene assays. The results show that RAW264.7 cells treated with GCWB1176 or LPS resulted in significantly increased iNOS protein expression and iNOS promoter luciferase activity, which was consistent with NO secretion. Our results suggest that GCWB1176 promoted the release of NO by upregulating the transcription of iNOS.

**Figure 6:**
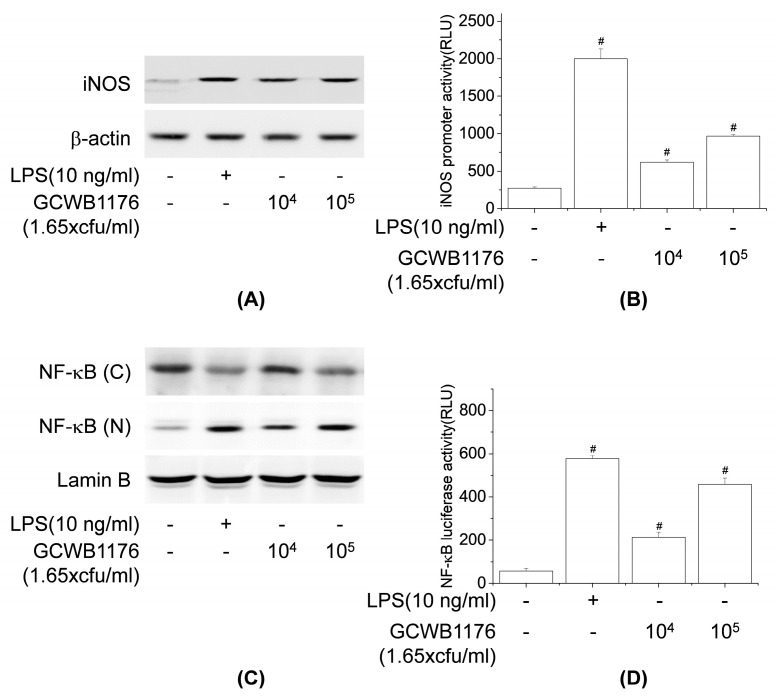
Effect of GCWB1176 on iNOS and NF-κB activation in macrophages. Cells were treated with LPS (10 ng/mL) or GCWB1176 for 24 or 1 h, and the protein levels of iNOS (**A**) and NF-κB p65 (**C**) were determined immunochemically using its specific antibody. The levels of β-actin and lamin B were measured as internal loading controls. Cells were transfected with piNOS-Luc (**B**) and pNF-κB-Luc (**D**) for 18 h, then treated with LPS (10 ng/mL) or GCWB1176 for 24 or 1 h. Cell lysates were assayed for luciferase activity. Luciferase activity was normalized to β-galactosidase activity. # *p* < 0.001 vs. control group.

NF-κB, the immune-related transcriptional factor, plays an important role in the production of specific cytokines [31]. Accordingly, we estimated whether GCWB1176 induces NF-κB activation using Western blotting and luciferase reporter gene assays. As shown in Figure 6C,D, GCWB1176 significantly enhanced the nuclear translocation of NF-κB-p65 and NF-κB promoter luciferase activity in a concentration-dependent manner. These results suggest that upregulation of NO and cytokines by GCWB1176 was through the NF-κB pathway in macrophages.

## 4. Discussion

The immunostimulatory potential of probiotics may be useful for the prevention and treatment of complex disorders ranging from diarrhea to allergy. Several LABs, including *Lactococcus*, *Enterococcus*, *Lactobacillus* and *Leuconostoc*, have been reported to have beneficial health effects by stimulating the immune system [19,20,21,22,23]. The immunostimulatory mechanism of LAB comprises regulation of the T cell effector subset, the improvement of humoral immunity and activation of macrophages and lymphocytes [32]. In the present study, we investigated the immunomodulatory properties of *Lactococcus lactis* GCWB1176 isolated from mozzarella cheese using mice splenocytes and a mouse model of CTX-induced immunosuppression.

Probiotics have recently become popular among natural products with immunostimulating effects. One important component for the development of probiotics as a health functional food is the presence of antibiotic resistance genes. Interestingly, many studies have shown that several species of LAB, including *S. thermophilus*, contain antibiotic-resistant genes or exhibit resistance to antibiotics, such as tetracycline, streptomycin chloramphenicol, gentamycin and kanamycin [33]. In this study, *Lactococcus lactis* subsp. *lactis*, isolated from mozzarella cheese, is a nonpathogenic Gram-positive bacterium, and it is the most commonly used cheese starter. GCWB1176 did not exert a hemolysis effect on cells compared to the positive control *E. coli* ATCC 25922. Although the EFSA cutoffs calculated here indicated that GCWB1176 does not show resistance against antibiotics, further studies are required to determine whether resistance genes can confer acquired resistance at a genetic level using PCR or whole genome sequencing.

CTX is commonly used to study immunomodulatory activity. CTX suppresses the immune system through inactivation of macrophages and lymphocytes and reduces the levels of inflammatory cytokines [11,12,13]. We investigated the immunostimulating effect of GCWB1176 in CTX-induced immunosuppressive mice. In our model, CTX reduced the spleen and thymus index in mice, which reflects non-specific immunity. Several studies have reported that immunomodulatory agents, including *Lactobacillus plantarum*, can restore spleen and thymus weight in CTX-induced immunosuppressive mice [34,35]. Similarly, we found that GCWB1176 orally administered for 16 consecutive days to CTX-treated mice increased spleen and thymus indices as compared to mice treated with CTX alone. The results also show that CTX markedly reduced the body weight. In addition, our results show that the spleen and thymus indices in GCWB1176 treatment groups were significantly higher than those in the CTX-treated group at 16 days, and the spleen and thymus indices were close to normal level at 16 days. These results suggest that GCWB1176 could resist the immunosuppressive effect on immune organ development.

Several authors have reported that immunostimulating agents including LAB could stimulate the production of proinflammatory cytokines and NK cell activity in cyclophosphamide-induced immunosuppressed animals [13,22,33]. Our results demonstrate that GCWB1176 induced the secretion of TNF-α, IFN-γ, IL-2, IL-4, IL-10 and IL-12p70 in mice with CTX-induced immunosuppression. In addition, our results demonstrate that GCWB1176 enhanced lymphocyte proliferative responses to T mitogen and NK cell activity in mice with CTX-induced immunosuppression.

Macrophages play an important role in host defense mechanisms and are an important part of the innate immune system. Many immunomodulators including LAB activate the immune response by activating macrophages [13,22,33]. The function of macrophages in innate immunity is initiating, propagating and performing phagocytosis against pathogens [29]. In addition, the activated macrophages produce a variety of immunomodulators including proinflammatory cytokines and NO [30]. NO, which is synthesized from l-arginine by inducible nitric oxide synthase, contributes to the killing of foreign pathogens and tumor cells and mediates a variety of biological functions as an intracellular messenger molecule [7]. Activated macrophages stimulate the production of various immunomodulatory cytokines, such as TNF-α, IL-6, IL-10 and IL-12 [35]. These immunomodulatory cytokines are involved in phagocytosis, inflammation, cell differentiation, proliferation, apoptosis and the promotion of immune cell functions [5,6]. In the present study, we found that GCWB1176 enhanced the phagocytic activity of RAW264.7 macrophages. In the present study, GCWB1176 increased the production of NO and macrophage-related cytokines TNF-α, IFN-γ, IL-1β, IL-4, IL-10 and IL-12p70 in mouse splenocytes and RAW264.7 macrophages, as well as enhanced iNOS protein expression and iNOS promoter luciferase activity in RAW264.7 macrophages. It was recently reported that the increase in immunomodulatory cytokines are associated with activation of NF-κB pathways [36]. In macrophages, NF-κB is an important transcriptional factor of immune activation that upregulates the expression of many cytokines [36]. It was recently reported that *Lactobacillus rhamnosus* GG, *Lactobacillus helveticus* IMAU70129, *Lactobacillus casei* IMAU60214 and *Lactobacillus plantarum* LM1004 induce NF-κB activation in human and mouse macrophages [37,38]. In the present study, we observed enhanced nuclear translocation of NF-κB and NF-κB promoter luciferase activity in RAW264.7 macrophages.

In summary, we evaluated the immunomodulatory effect of GCWB1176 in mouse primary splenocytes and RAW264.7 macrophages. GCWB1176 enhanced the phagocytic ability of macrophages, increased NK cell activity and increased the expression of immune modulators such as NO and cytokines. In addition, our results show that the NF-κB signaling pathways are responsible for these effects. More importantly, GCWB1176 improved spleen and thymus indices in CTX-induced immunosuppressed mouse model. These findings suggest that *Lactococcus lactis* GCWB1176 could function as an effective immunostimulatory agent in early innate immune responses.

## Figures and Tables

**Table 1:** Hemolysis activity.

	Strain	Positive		Negative
		Alpha	Beta	Gamma
Control	*Escherichia coli* ATCC25922	O		
	*Staphylococcus aureus* ATCC12600		O	
	*Enterococcus faecalis* ATCC19433			O
Test	*Lactococcus lactis* GCWB1176			O

**Table 2:** Minimum inhibitory concentration (MIC) of *Lactococcus lactis* GCWB1176.

Antibiotic Resistance Test									
Strain	Minimum Inhibitory Concentration (mg/L) of Antibiotics								
	Amp	Ery	Gen	Tet	Str	Van	Chl	Kan	Cli
GCWB1176	<1	<0.2	≤4	<0.2	≤32	<1	≤6	≤12	<0.1
EFSA breakpoint	2	1	32	4	32	4	8	64	1

Amp, ampicillin; Ery, erythromycin; Gen, gentamicin; Tet, tetracycline; Str, streptomycin; Van, vancomycin; Chl, chloramphenicol; Kan, kanamycin; Cli, clindamycin.
